# Flavour distribution and release from gelatine-starch matrices

**DOI:** 10.1016/j.foodhyd.2020.106273

**Published:** 2021-03

**Authors:** Katy Su, Marine Brunet, Daniel Festring, Charfedinne Ayed, Tim Foster, Ian Fisk

**Affiliations:** a The University of Nottingham, Division of Food, Nutrition and Dietetics, School of Biosciences, Sutton Bonington Campus, Loughborough, LE12 5RD, UK; b VetAgro Sup, Campus Agronomique, Lempdes, 63370, France; c Nestlé Product Technology Centre Confectionery, P.O. Box 204, Haxby Road, York, YO91 1XY, UK

**Keywords:** Microstructure, Gels, Phase separation, Aroma release

## Abstract

Microstructure design of protein-polysaccharide phase separated gels has been suggested as a strategy to nutritionally improve food products. Varying the phase volumes of a phase separated matrix may affect texture and overall flavour balance of the final product, which are both important for consumer acceptance. The aims of this study were to investigate how modifying the phase volumes of a gelatine-starch biphasic mixture affected aroma release, and how addition of sucrose affects phase separation, flavour distribution and aroma release.

Biphasic gels of different microstructures with the same effective concentration of gelatine and starch in each phase were developed. Microstructure significantly affected aroma release *in vitro* but not *in vivo* when panellists (n = 5) chewed and swallowed the sample. Addition of sucrose (0–60%) to the biphasic mixture significantly reduced water activity, affected the microstructure and affected aroma distribution in each phase and subsequent release rates depending on the physicochemical properties of the aroma volatile. In general, affinity for the gelatine phase for the less hydrophobic, more volatile compounds was not significantly affected by sucrose concentration. Whereas an increased affinity for the starch phase for the more hydrophobic, less volatile compounds was observed with increased sucrose as the starch phase becomes more dispersed at sucrose concentrations between 40 and 60%. The results of this study may be of interest to researchers and industry to enable prediction of how reformulation, such as reduction of sucrose, to meet nutritional guidelines may affect the overall aroma balance of a phase separated food matrix.

## Highlights

• Aroma affinity to gelatine or starch depends on physicochemical properties.
• Changes in sucrose concentration affects aroma release rates from gels.
• Sucrose concentration affects microstructure of gelatine-starch gels.
• Microstructure changes result in changes in overall aroma balance.

## Graphical abstract

**Figure:**
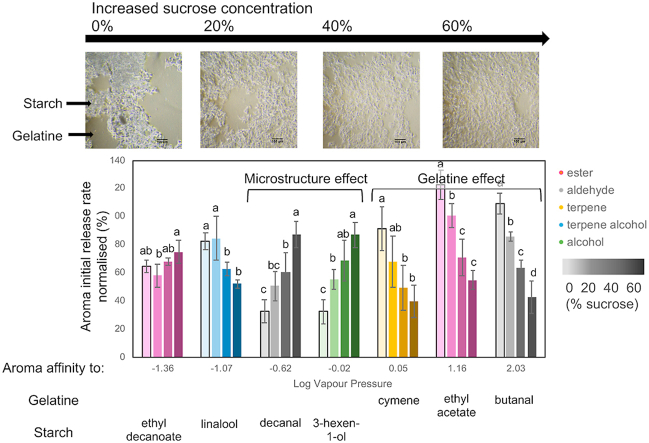

## 1 Introduction

Foods often contain a mixture of hydrocolloids to provide synergistic effects. Thermodynamic incompatibility of the hydrocolloids may lead to phase separation of the hydrocolloids into a phase rich in one hydrocolloid dispersed in the other. Confectionery gels typically contain the hydrocolloids gelatine, starch or pectin (Burey, Bhandari, Rutgers, Halley, & Torley, 2009). Depending on whether gelatine or starch is the continuous phase, achieved by modifying the phase volumes (Foster, Brown, & Norton, 1995), different textures are produced (Groves, 2003). Also, addition of starch to gelatine gels resulted in rigid gels (Piccone, Rastelli, & Pittia, 2011), compared to a melt-in-mouth texture associated with pure gelatine gels. Understanding protein-polysaccharide phase separation will enable finer control of microstructure in food applications such as confectionery gels. Furthermore, microstructure design strategies have been shown to be effective in reducing tastant concentration for nutritional purposes without affecting flavour perception (Stieger & van de Velde, 2013). These structural modifications are likely to affect the overall flavour balance of a product. Although aroma release from single hydrocolloid matrices is well understood, there is limited understanding of aroma distribution and release from mixed hydrocolloid matrices (Savary, Lafarge, Doublier, & Cayot, 2007; Taylor, Besnard, Puaud, & Linforth, 2001) and microstructure design rules are yet to be developed.

Microstructures and textures of gelled products occur from competing processes: phase separation and gelation, which occur simultaneously (Lorén et al., 2001; van de Velde, Hoog, Oosterveld, & Tromp, 2015). Textural properties and microstructure of phase separated gelatine-starch gels have been characterised (Brown, Foster, & Norton, 1995; Firoozmand & Rousseau, 2013; Marfil, Anhê, & Telis, 2012) however the impacts on flavour release is less understood. Only one study (Taylor et al., 2001) so far in literature has investigated the link between phase separated gels and aroma release. Release of compounds with different physicochemical properties were investigated *in vivo*, and no significant difference in maximum intensity of release and time to maximum intensity was observed between the gels with different microstructures (Taylor et al., 2001).

Factors that affect flavour release from these matrices include interactions of flavour compounds with the hydrocolloids, and the effects of the hydrocolloids on diffusion rate of the flavour compounds to the food-saliva and food-air interface (Harrison & Hills, 1996). Interactions and affinity for the hydrocolloids depend on physicochemical properties of the compounds such as hydrophobicity and volatility, for example more hydrophobic compounds tend to be less volatile and are less likely to be released from a hydrocolloid based matrix (Boland, Delahunty, & van Ruth, 2006; Zafeiropoulou, Evageliou, Gardeli, Yanniotis, & Komaitis, 2012). Hydrophobic compounds tend to have long hydrophobic chains, and studies have shown greater interactions with starch (Golovnya, Terenina, Krikunova, Yuryev, & Misharina, 2001; Heinemann, Conde-Petit, Nuessli, & Escher, 2001) and proteins (Reiners, Nicklaus, & Guichard, 2000) with increased chain length.

Aroma volatiles may interact with proteins through non-polar interactions, and aldehydes can undergo cysteine-aldehyde condensation reactions and Schiff base formation with amino groups of proteins (Tromelin, Andriot, & Guichard, 2006). Proteins have been shown to retain aroma volatiles *in vitro* using headspace measurements, however *in vivo* measurements have shown certain compounds are unaffected by presence of proteins, suggesting dilution effects by saliva and also interactions between flavour and mouth tissue (Tromelin et al., 2006).

Acid thinned starch is typically used in confectionery as it has a lower hot paste viscosity (Rapaille & Vanhemelrijck, 1997), achieved by partial hydrolysis and reducing the molecular weight without disrupting the integrity of the uncooked granule (Burey et al., 2009). Starch can affect flavour release by forming complexes with aroma, crystallisation of these complexes, and on a larger scale - structured network formation through aggregation or phase separation (van Ruth & King, 2003). Starch-aroma interactions have been widely reported in literature, with many studies focusing on the structure of starch-aroma inclusion complexes and aroma release (Arvisenet, Voilley, & Cayot, 2002; Conde-Petit, Escher, & Nuessli, 2006; Escher, Nuessli, & Conde-Petit, 2000; Nuessli, Sigg, Conde-Petit, & Escher, 1997). Aroma volatiles interact with amylose helices either inside the hydrophobic cavity of the helix or in between the free space of the helices (Escher et al., 2000). Studies have also reported aroma binding to amylopectin (Arvisenet et al., 2002; van Ruth et al., 2003) though not through inclusion complexes since amylopectin is mainly branched, but through hydrogen bonds. Furthermore the concentrations of gelatine and starch in the food matrix may also affect texture, and the generation of fresh surfaces upon gel breakdown during mastication (Morris, 1993) which influences mass transfer of flavour compounds (Harrison et al., 1996).

To control the microstructure of a phase separated hydrocolloid network, phase diagrams are used as they provide information about the minimum concentrations of the hydrocolloids that are required to form a phase separated network, and also the concentrations to have a selected hydrocolloid as the continuous phase (Foster et al., 1995; Norton & Frith, 2001). These can be constructed using the phase-volume ratio method outlined by Lundin, Williams, and Foster (2003) and Polyakov, Grinberg, and Tolstoguzov (1980). On a phase diagram, a binodal separates regions of concentrations where the different hydrocolloids are miscible, from the concentrations in which the hydrocolloids phase separate. These segregative interactions can occur when there is electrostatic repulsion between hydrocolloids of the same charge, or if they have no charge then phase separation is most likely a result of thermodynamic incompatibility (Syrbe, Fernandes, Dannenberg, Bauer, & Klostermeyer, 1995). A phase diagram is unique to the type of hydrocolloids used and also other factors such as the pH of the mixture and temperature. Addition of a solute, such as sucrose to the mixture affects phase separation by affecting the thermodynamic compatibility of the hydrocolloids and hence the phase diagram (Schorsch, Jones, & Norton, 2000; Spyropoulos, Portsch, & Norton, 2010).

The presence of sugar in the food matrix also affects gelation properties of gelatine and polysaccharides differently, the texture of a product and aroma release (Ellis, Mills, Norton, & Norton-Welch, 2019; Hansson, Andersson, & Leufvén, 2001; Kasapis, Al-Marhoobi, Deszczynski, Mitchell, & Abeysekera, 2003; Spyropoulos et al., 2010). Sugar delays gelatinisation of starch as it lowers the water activity, and stabilises the amorphous regions through formation of bridges between chains (Spies & Hoseney, 1982). Increased concentrations of sucrose increased the number of junction zones and decreased the size of these junction zones in polysaccharide (Nishinari et al., 1992) and gelatine (Oakenfull & Scott, 1986) gels. At low sucrose concentrations (~33% sucrose), the temperature of gelation is affected and there is greater cross-linking of both hydrocolloids forming a stronger network (Kasapis et al., 2003). Intermediate levels of sucrose (40–60% sucrose) affects gelatine differently to polysaccharides, where an increase in cross-linking of gelatine resulting in a gel with greater network strength and a decrease in gel strength and disaggregation of polysaccharides was observed (Kasapis et al., 2003). Less free water is available to provide thermodynamic stability for an ordered polysaccharide network (Ellis et al., 2019; Kasapis, Mitchell, Abeysekera, & MacNaughtan, 2004) and the increase in viscosity of the network reduces the mobility of the chains to aggregate, therefore helix nucleation occurs but less aggregates (Normand et al., 2003). Increased sucrose concentrations up to 60% has been shown to increase the release of aroma volatiles into the headspace from a soft drink model system (Hansson et al., 2001). Also both salting-in and salting-out effects on compounds with different physicochemical properties was observed in a simple aqueous model system with increased sucrose concentrations (Friel, Linforth, & Taylor, 2000). At higher concentrations of sucrose (>70%), there is an increased viscoelasticity of polysaccharide sugar mixtures and a slow build-up of the polysaccharide network (Kasapis et al., 2003; Kasapis et al., 2004).

The impact of hydrocolloid type on partition coefficients is well understood from gelatine and starch matrices when the hydrocolloids are separate. Also Taylor et al. (2001) showed the effects of different microstructures on aroma release. However, to the best of the authors knowledge there are no studies that link controlling the microstructure of biphasic mixtures with the same effective concentration of both hydrocolloids in each phase to flavour release which is a key element of consumer acceptance. Therefore two aspects are considered in this study. Firstly, the effects of modifying phase volumes of gelatine and starch biphasic gels, with the same effective concentration of hydrocolloids in each phase, on aroma release *in vitro* and *in vivo* is investigated to understand thermodynamic and kinetic factors driving release. Also, the effects of sucrose as an additional component to the biphasic gel, on flavour release and distribution was measured. Linking phase separation with flavour release and distribution, enables prediction of how reduction of tastants such as sucrose from a protein-polysaccharide food matrix will affect the overall flavour balance of a reformulated product.

## 2 Materials and methods

### 2.1 Phase separation measurements

Gelatine (240 bloom, type A, mmingredients, UK) and acid thinned starch (GPC, Iowa, USA) was dissolved in water at 60 °C, and 95 °C respectively, with continuous stirring throughout the experiment. Stock solutions of 25%w/v gelatine with 10%w/v starch and 20%w/v gelatine with 8%w/v starch were prepared and the varying proportions of the stock solutions that were mixed together to form the final solution are listed in Table 1. The solutions were incubated at 60 °C for 24 h to observe phase separation. The phase separated layers were measured by weight and used to produce a phase diagram according to the phase-volume-ratio method (Lundin et al., 2003; Polyakov et al., 1980).

**Table 1:** Concentrations of hydrocolloids used to produce phase diagram.

	Gelatine		Starch	
Stock solution (%)	1.25%	2.20%	1.10%	2.8%
Mixing ratio of stock solution (gelatine:starch)	Concentration of stock solution in final solution (%w/v)			
1:9	10		90	
2.5:7.5	25		75	
5:5	50		50	
7.5:2.5	75		25	
9:1	90		10	

### 2.2 Preparation of biphasic gelatine-starch gels

Stock solutions were prepared in the same way as section 2.1. According to the tie-lines on the phase diagram, concentrations to produce a gelatine or starch continuous and bicontinuous gel were selected. The relative proportion of appropriate stock solutions was taken and mixed together and incubated at 60 °C to allow the hydrocolloids to phase separate. Preliminary experiments showed after 2 h, no change in phase separated structure was observed. After 2 h, 5%w/v aroma consisting of 150 ppm ethyl butyrate, ethyl acetate, ethyl hexanoate and ethyl octanoate in water was mixed in. The mixture was poured into cube shaped molds for 1 cm^3^ individual gel sizes and quenched to −80 °C. Samples were equilibrated to 20 °C before testing.

### 2.3 Preparation of phase separated solutions and gels with sucrose or maltodextrin

0, 20, 40, 60%w/v sucrose was dissolved in water, and 10%w/v of the same gelatine and acid thinned starch as above were added to separate sucrose solutions and stirred at 60 °C and 95 °C respectively. To test a hypothesis using mixtures with additional maltodextrin, 5 and 10%w/v maltodextrin DE6 was added to the starch solutions with no sucrose. 5 ml of each solution was mixed together, and 1 ml of an aroma solution dissolved in water (Table 2) was added. Preliminary trials showed many of the aroma compounds were not stable after bulk phase separation of the biopolymer solution at 60 °C in a water bath; hence, the solution was mixed and left for 30 min then centrifuged at 3000 RPM for 5 min at 40 °C to obtain a phase separated solution. For the gels, the phase separated solution was mixed and poured into cube shaped molds for 1 cm^3^ individual gel sizes and quenched to −80 °C. Samples were equilibrated to 20 °C before testing.

**Table 2:** Aroma volatiles, physicochemical properties (25 °C) values from EPI Suite.

Compound	Molecular weight (g/mol)	Log P	Log Vapour Pressure
Ethyl acetate	88.11	0.86	1.99
Ethyl butyrate	116.16	1.85	1.16
Ethyl hexanoate	144.21	2.83	0.26
Ethyl octanoate	172.26	3.81	−0.63
Ethyl decanoate	200.32	4.79	−1.37
Isoamyl acetate	130.18	2.26	0.75
α-pinene	136.23	4.27	0.60
*p*-cymene	134.22	4	0.06
d-limonene	136.23	4.83	0.16
2-methyl-butan-1-ol	88.15	1.26	0.66
3-hexen-1-ol	100.16	1.61	−0.03
Linalool	154.25	3.38	−1.08
Geraniol	154.25	3.47	−1.70
Butanal	72.11	0.82	2.03
Hexanal	100.16	1.8	0.98
Octanal	128.21	2.78	0.17
Decanal	156.26	3.76	−0.63
Benzaldehyde	106.12	1.71	0.004
γ-decalactone	170.25	2.57	−2.29

### 2.4 Water activity measurements

A_w_ (water activity) of the samples was measured using an AQUALAB water activity meter (Labcell, UK). 2 ml of each of the gelatine or starch phases with different sucrose concentrations was pipetted into a clear container for analysis at 25 °C.

### 2.5 Microscopy

Samples were pipetted onto microscope slides with a cover slip. Phase separated microstructures of biphasic gelatine-starch mixtures were taken on an EVOS f1 inverted microscope (Fisher Scientific, UK). Phase contrast microscopy on a Nikon Eclipse Ci microscope (Nikon, Japan) was used to observe microstructure changes upon addition of sucrose.

### 2.6 Texture analysis

Texture profile analysis (TPA) was carried out on the samples using a TA. TX Plus Texture Analyser (Stable Micro Systems Ltd., Surrey, UK). 1 cm^3^ gel samples were compressed using a 2 mm cylindrical probe according to the conditions described by Delgado and Bañón (2015), except a 0.01 N trigger force was used. Hardness (peak force during the double compression) was evaluated for the samples.

### 2.7 Aroma release from biphasic gelatine-starch gels

Phase separated gelatine-starch gels were melted in a sealed glass jar whilst stirring (250RPM) at physiological temperatures (37 °C) and aroma released into the headspace was continuously sampled through a heated interface into the atmospheric pressure chemical ionisation – mass spectrometry (APCI-MS) source (MicroMass, Manchester, UK). This produced aroma release curves that increased as the gel was melting then plateau when equilibrium was reached. The initial rate was taken from the linear portion up to 30s and values were normalised to the maximum intensity of release. Four esters: ethyl acetate, ethyl butyrate, ethyl hexanoate and ethyl octanoate were measured using selected ion monitoring analysis mode (protonated ions 89, 117, 145 and 173 m/z).

Experiments which involved panellists has been approved by the Biosciences Ethical Committee at the University of Nottingham. In-nose measurements of the four esters was taken as panellists (n = 5) chewed the sample freely, then swallowed the sample. Five panellists were selected since it has been shown that five panellists consuming three replicates provides a representative measure of aroma release (Yang et al., 2011). Panellists recorded approximately each chew of the sample. Aroma per chew was calculated from the total area under curve of the release curve divided by the chew number.

### 2.8 Aroma distribution and release from gelatine-starch-sucrose gels and solutions

The supernatant (gelatine phase) and the sediment (starch phase) was pipetted into separate GC-MS headspace vials and weighed. 30 μl 0.01% 3-heptanone (Sigma Aldrich, UK) in methanol (Fisher Scientific, UK) was added, and the vials sealed for analysis. Samples were analysed in a randomised order, in triplicate. Analysis was carried out using a Trace 1300 Gas Chromatography, Single-Quadruple Mass Spectrometer (Thermo Fisher Scientific, UK). Samples were incubated at 60 °C for 30 min prior to solid phase micro extraction (SPME) of 19 aroma volatiles (Table 2). The SPME fibre (50/30 μm, DVB/CAR/PDMS, Sigma Aldrich, UK) extracted for 15 min, then thermally desorbed at 250 °C for 2 min. A 30 m ZB-WAX capillary column was used with a 0.25 mm internal diameter and 1 μm film thickness (Phenomenex, UK). Oven temperature was maintained at 50 °C for 2 min then ramped to 250 °C at 6 °C/min and the MS operated in full scan mode from 35 to 300 m/z with a scan time of 0.2s. The peak area of the aroma compounds was compared to the peak of the internal standard 3-heptanone, and corrected for the weight of the sample. Aroma distribution ratios were calculated from the concentration in the headspace of the gelatine phase divided by the sum of the concentration in the gelatine and starch phases. A value between 0.5 and 1 indicated greater affinity for the gelatine phase, and 0–0.5 for the starch phase.

Aroma release rates from gels with sucrose were measured as described in section 2.7, except the MH^+^ or [M-H_2_O + H]^+^ (Taylor et al., 2001) ions measured were 73, 83, 117, 134, 138, 157, 201 m/z.

### 2.9 Sucrose analysis

10 μl of the supernatant and sediment was added to separate solutions of 3 ml 50:50 water and methanol (Fisher Scientific, UK) solvent. Samples were left on a roller mixer for 60 min and 1 ml of the solution was taken for liquid chromatography mass spectrometry (LC-MS) analysis. The LC-MS (1100 Series, Agilent) with a degasser (G1322A, Agilent), pump (G1312A, Agilent) and auto-sampler (G1313A, Agilent), interfaced with a Quattro Ultima mass spectrometer (Micromass, UK Ltd.) was used for the analysis of sucrose. Sucrose (341.3 m/z) was measured in a negative ionisation selected ion mode. A Luna 5 μm NH_2_ 100 A column (250 × 3.2 mm, 5 μm, Phenomenex) was used, and the volume injected was 5 μl at a flow rate of 0.7 ml/min 80% acetonitrile, 20% water. The concentration of sucrose (at 341.3 m/z) was calculated using a sucrose standard curve (61.5–500 mg/mL).

### 2.10 Statistical analysis

All statistical tests were performed using XL STAT (Addinsoft, NY, USA). Statistical differences between samples (α = 0.05, p-values < 0.05) were tested using analysis of variance (ANOVA) and Tukey's HSD post-hoc tests. Aroma compounds were grouped based on dissimilarity agglomerative hierarchical clustering (AHC), isoamyl acetate and hexanal were moved to a different group based on a higher similarity in behaviour to the group.

## 3 Results and discussion

### 3.1 Gelatine – starch phase diagram

**Fig. 1:**
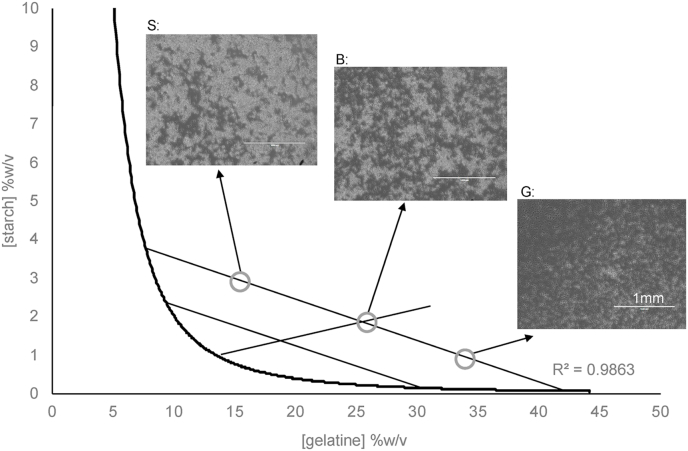
Phase diagram of a gelatine – acid thinned mixture (60 °C). Different microstructures observed include S: a starch dominating structure (light aggregates) with gelatine (dark clear phase) dispersions, B: a bicontinuous structure and G: a gelatine dominating structure with starch aggregate dispersions. Scale bars = 1 mm.

A phase diagram was produced using the well established phase-volume-ratio method (Lundin et al., 2003; Polyakov et al., 1980) for this specific gelatine – starch mixture to enable control of the microstructure. It is known that protein-polysaccharide mixtures show asymmetry on a phase diagram, where a lower concentration of polysaccharide is required for phase separation compared to proteins (Morris, 1990), and this is observed in Fig. 1. Tie-lines connect different initial concentrations that phase separate to the same final effective concentration which is the point of intersection with the binodal (Morris, 1990; Norton et al., 2001). For example the three concentrations circled on Fig. 1 have different microstructures, however, the effective concentration in the protein phase and the polysaccharide phase is the same. The light phase observed in Fig. 1 is the aggregated starch phase (Abeysekera & Robards, 1995), and dark phase is the clear gelatine phase. A starch dominating structure is observed in Fig. 1S, with gelatine rich regions dispersed, and vice versa in Fig. 1G. The bicontinuous structure in Fig. 1B is around the phase inversion point, on the rectilinear diameter which intersects the tie-lines at the midpoint.

### 3.2 Aroma release from biphasic gelatine-starch gels

Aroma release was investigated from gels with microstructures representing starch continuous (S), bicontinuous (B), and continuous (G) structures (Fig. 1). Table 3 shows the effects of gel structure on release of linear esters with a range of hydrophobicity values. There are two aspects to consider, the effects of gel structure on release of aroma volatiles and the effect of ester chain length and hydrophobicity on their release. Firstly, if we compare the different gel microstructures, time to reach maximum release of aroma (Tmax) from G is significantly slower than S by a minimum of 20s for each aroma compound except ethyl octanoate (P < 0.05). Since gel S is significantly softer than G, this resulted in a faster rate of dissolution of the gel, hence Tmax was faster (Table 3). Also, variations in the affinity of the volatiles for the dominating phase may also affect release into the headspace during dissolution. This is described in more detail in section 3.4.

**Table 3:** Aroma release from biphasic gelatine-starch gels with different microstructures. G = gelatine continuous, B = bicontinuous, S = starch continuous. Superscript letters indicate significant differences (P < 0.05) within each group of parameters (e.g. Tmax, Imax) for all compounds.

	Microstructure		
	G	B	S
**Hardness (N)**	1.42^a^ ± 0.18	0.74^b^ ± 0.03	0.38^c^ ± 0.01
pH	3.99^a^ ± 0.01	3.96^a^ ± 0.03	3.98^a^ ± 0.02
***in vitro***			
**T**_**max**_**(min)**			
Ethyl acetate	1.9^ab^ ± 0.3	1.4^bc^ ± 0.2	1.3^c^ ± 0.2
Ethyl butyrate	1.9^ab^ ± 0.3	1.6^bc^ ± 0.3	1.3^c^ ± 0.2
Ethyl hexanoate	1.9^ab^ ± 0.4	1.6^bc^ ± 0.2	1.5^bc^ ± 0.2
Ethyl octanoate	2.3^a^ ± 0.3	2.4^a^ ± 0.1	2.3^a^ ± 0.1
**Dissolution time (min)**	1.7^a^ ± 0.2	1.4^a^ ± 0.1	1.1^b^ ± 0.1
***in vivo***			
**Aroma per chew (peak area units)**			
88	10835^ab^ ± 2470	10869^ab^ ± 1879	13122^a^ ± 2789
116	9215^bcd^ ± 1430	8762^bcd^ ± 1659	11278^ab^ ± 1342
144	10075^abc^ ± 1277	10422^abc^ ± 1167	12638^ab^ ± 1926
172	5939^d^ ± 1197	6041^d^ ± 751	7041^d^ ± 1225
**I**_**max**_**(ppbv)**			
88	34^a^ ± 13	31^a^ ± 7.2	36^a^ ± 12
116	20^b^ ± 5.7	18^b^ ± 2.5	20^b^ ± 4.6
144	6.4^c^ ± 1.8	6.1^c^ ± 1.3	7.6^c^ ± 1.8
172	1.3^c^ ± 0.4	1.4^c^ ± 0.2	1.5^c^ ± 0.5
**T**_**max**_**(min)**			
88	0.42^a^ ± 0.05	0.44^a^ ± 0.08	0.40^a^ ± 0.04
116	0.40^a^ ± 0.06	0.42^a^ ± 0.07	0.38^a^ ± 0.03
144	0.39^a^ ± 0.06	0.42^a^ ± 0.06	0.40^a^ ± 0.04
172	0.47^a^ ± 0.05	0.48^a^ ± 0.06	0.43^a^ ± 0.04
**Number of chews**	19^a^ ± 2	18^a^ ± 2	16^a^ ± 2

Secondly, comparing the different compounds, it takes significantly longer for ethyl octanoate (P < 0.05) to reach Tmax in all three gel structures by more than 20s compared to other compounds. Despite it being a hydrophilic matrix, there was a greater release of the hydrophilic compounds compared to the hydrophobic. Therefore, it is not just hydrophobicity affecting the interactions, taking into account the volatility of the compounds is also important to understand trends in Tmax and maximum intensity of release (Imax). This agrees with previous research that showed esters with greater chain lengths have a lower air/gel partition coefficient, hence were less likely to be released (Boland et al., 2006; Zafeiropoulou et al., 2012).

No effect of microstructure on release parameters was observed *in vivo,* similar to observations made in phase separated gels without control of microstructure (Taylor et al., 2001). The aroma released per chew, Imax and Tmax was not significantly different (P > 0.05) for the three different gels (Table 3). Similar to dynamic release measured *in vitro*, significantly less (P < 0.05) ethyl octanoate was released into the nose space compared to ethyl acetate. Also Imax of the compounds follows the trend that the less hydrophobic, more volatile compounds reach a higher maximum intensity of release than the more hydrophobic ones. No significant differences (P > 0.05) in Tmax were observed *in vivo* between samples with different microstructures nor between the different compounds. Van Ruth and King (2003) also observed that *in vivo* no effect of starch concentration, or chain length on aroma release was observed, when differences were observed *in vitro.* The authors concluded that kinetic factors are more important than thermodynamic factors in determining release. Malone and Appelqvist (2003) showed that for starch gelled particles, breakdown by amylase is a key influencer of release rate alongside fracture properties. For gelatine gelled particles and gels, concentration and rate of heat transfer, which affects melting of the gel, influences release kinetics (Harrison et al., 1996; Malone et al., 2003). Therefore although differences were not observed for this particular gelatine-starch protein-polysaccharide matrix, for matrices with different hydrocolloids, modifying the microstructure may show a difference *in vivo* for hydrocolloids that aren't affected by amylase and with melting points less than physiological temperatures.

Since a free chewing protocol was implemented, variations in chewing patterns and oral physiology (Blissett, Hort, & Taylor, 2006) contribute to these effects observed. Though, there was no significant difference (P < 0.05) in chew number, which would affect the surface area generated per chew for faster mass transfer of volatiles (Hills & Harrison, 1995; Morris, 1993) hence similar release parameters were observed. Physicochemical properties of compounds affect timings of release under simple conditions *in vitro,* but not in more complex conditions *in vivo* when other factors such as chewing and dilution with saliva occurs. (Baek, Linforth, Blake, & Taylor, 1999; Hollowood, Linforth, & Taylor, 2002; Lethuaut, Weel, Boelrijk, & Brossard, 2004; Weel et al., 2002).

### 3.3 Effect of sucrose on phase separation of gelatine-starch mixtures

**Fig. 2:**
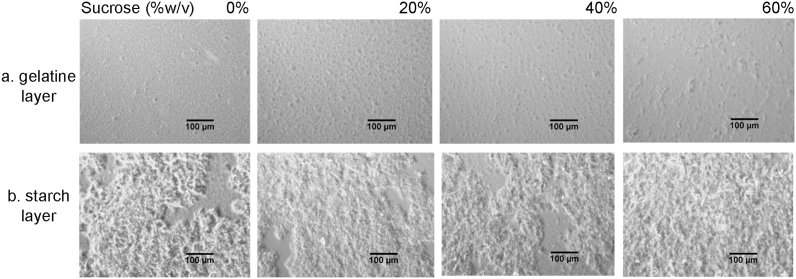
Effects of sucrose on microstructure of the gelatine (a) and starch (b) phase separated layers, gelatine = clear region, starch = aggregated structure.

Since food matrices are not as simple as two hydrocolloids in solution, the effects of addition of sucrose on phase separation and aroma release was investigated. However, the specific effects of sucrose on the phase diagram were not studied since previous literature has already described these effects (Spyropoulos et al., 2010). Instead the focus of the rest of this study is on the changes in microstructure and how that impacts flavour release. Gelatine and starch bulk phase separated into layers that were gelatine continuous with starch aggregates (Fig. 2a), and starch continuous with gelatine dispersions (Fig. 2b). As described in section 3.2, amylose from corn starch is known to form an aggregated structure (Abeysekera et al., 1995). Complete demixing did not occur after centrifugation as both polymers are present in the gelatine rich and starch rich layers. Addition of sucrose affects gelatine and polysaccharides differently at certain concentrations (Kasapis et al., 2003). The less aggregated starch structure observed as a result of increased sucrose levels in Fig. 2b is similar to what was observed for other polysaccharides at these concentrations of sucrose (Kasapis et al., 2003; Normand et al., 2003). The effect on gelatine is less clear, in terms of the microstructure in Fig. 2a, though Oakenfull and Scott (1986) showed that increased sucrose concentrations resulted in an increase in rigidity of gelatine mixtures and Kasapis et al. (2003) further showed the separation into sugar rich and gelatine rich phases. Similar effects were observed in polysaccharide mixtures at sucrose concentrations up to 40%, however the authors showed that at concentrations between 40 and 60% sucrose, there is a transformation from a highly aggregated polysaccharide structure, to a less aggregated one as a reduction in free water reduces the ability for the polysaccharide network to form.

### 3.4 Effects of sucrose on flavour distribution

**Fig. 3:**
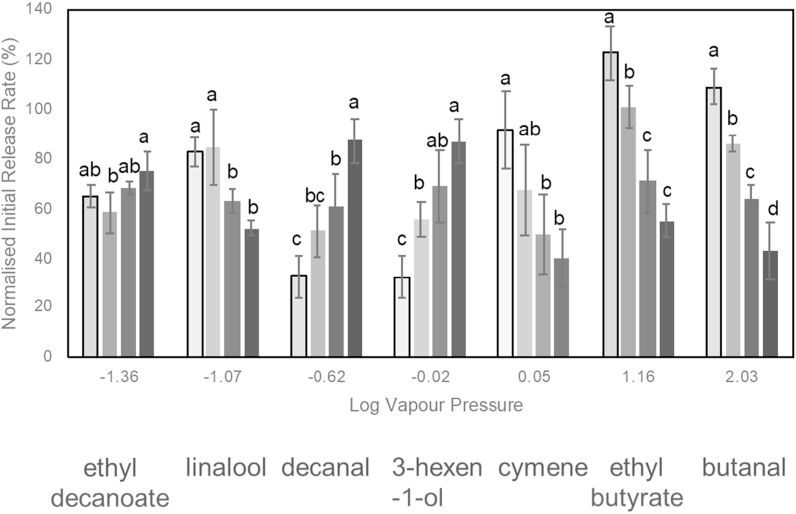
Aroma release rates from phase separated gelatine-starch gels with increased levels of sucrose (light to dark, 0% 20% 40% 60%). Different letters indicate a significant effect (P < 0.05) of sucrose on the release kinetics of that compound.

Aroma affinity for the gelatine or starch phase differed based on their physicochemical properties (Table 4) and compounds with similar behaviour was grouped using AHC (Fig. 3). Most studies have shown that release from starch gels is greater than gelatine gels (Boland, Buhr, Giannouli, & van Ruth, 2004; Kälviäinen, Roininen, & Tuorila, 2000; Piccone et al., 2011; Zhang & Barringer, 2018). Despite this, release was greater of some compounds from the gelatine phase, showing greater affinity for the gelatine phase in a mixed phase matrix, and also that understanding release from a single hydrocolloid matrix may not be directly applicable to a mixed hydrocolloid matrix.

**Table 4:** Phase properties and flavour distribution in phase separated gelatine-starch matrices with sucrose. Different letters indicate significant difference (P < 0.05) within each row of data. Aroma volatiles are divided into groups determined by agglomerative hierarchical clustering based on dissimilarity.

	Sucrose concentration % (w/v)			
	0	20	40	60
Water activity				
Gelatine phase	0.998 ^a^ ± 0.002	0.983 ^b^ ± 0.002	0.971 ^c^ ± 0.005	0.949 ^d^ ± 0.003
Starch phase	0.997 ^a^ ± 0.004	0.973 ^b^ ± 0.003	0.968 ^b^ ± 0.001	0.954 ^c^ ± 0.001
**Sucrose distribution ratio**	–	0.58 ^a^ ± 0.04	0.51 ^a^ ± 0.08	0.52 ^a^ ± 0.05
**Aroma distribution ratios**				
**Group A: affinity for gelatine, not affected by sucrose**				
Ethyl acetate	0.53 ^a^ ± 0.04	0.59 ^a^ ± 0.04	0.56 ^a^ ± 0.03	0.59 ^a^ ± 0.03
Ethyl butyrate	0.62 ^a^ ± 0.03	0.68 ^a^ ± 0.04	0.58 ^a^ ± 0.05	0.67 ^a^ ± 0.02
Isoamyl acetate	0.68 ^a^ ± 0.02	0.72 ^a^ ± 0.03	0.68 ^a^ ± 0.04	0.69 ^a^ ± 0.02
Butanal	0.52 ^a^ ± 0.03	0.57 ^a^ ± 0.01	0.50 ^a^ ± 0.004	0.49 ^a^ ± 0.04
Hexanal	0.54 ^a^ ± 0.03	0.56 ^a^ ± 0.05	0.54 ^a^ ± 0.03	0.52 ^a^ ± 0.03
**Group B: affinity for starch, both affected and not by sucrose**				
Ethyl hexanoate	0.50 ^a^ ± 0.03	0.51 ^a^ ± 0.02	0.39 ^ab^ ± 0.04	0.31 ^b^ ± 0.06
Ethyl octanoate	0.47 ^a^ ± 0.01	0.39 ^ab^ ± 0.07	0.29 ^bc^ ± 0.05	0.22 ^c^ ± 0.05
Octanal	0.44 ^a^ ± 0.03	0.46 ^a^ ± 0.06	0.34 ^ab^ ± 0.06	0.26 ^b^ ± 0.04
Decanal	0.42 ^ab^ ± 0.05	0.48 ^a^ ± 0.07	0.32 ^ab^ ± 0.03	0.25 ^b^ ± 0.06
Benzaldehyde	0.41 ^a^ ± 0.02	0.35 ^a^ ± 0.11	0.38 ^a^ ± 0.05	0.30 ^a^ ± 0.05
Linalool	0.34 ^a^ ± 0.03	0.40 ^a^ ± 0.07	0.35 ^a^ ± 0.07	0.27 ^a^ ± 0.04
Geraniol	0.41 ^a^ ± 0.03	0.47 ^a^ ± 0.11	0.39 ^a^ ± 0.07	0.31 ^a^ ± 0.06
2-methyl-butan-1-ol	0.35 ^a^ ± 0.01	0.39 ^a^ ± 0.07	0.35 ^a^ ± 0.06	0.32 ^a^ ± 0.04
3-hexen-1-ol	0.28 ^a^ ± 0.002	0.34 ^a^ ± 0.07	0.32 ^a^ ± 0.07	0.26 ^a^ ± 0.04
**Group C: affinity for gelatine, affected by sucrose**				
Ethyl decanoate	0.82 ^a^ ± 0.03	0.64 ^ab^ ± 0.15	0.49 ^ab^ ± 0.08	0.47 ^b^ ± 0.11
γ-decalactone	0.75 ^a^ ± 0.01	0.66 ^ab^ ± 0.11	0.46 ^b^ ± 0.06	0.43 ^b^ ± 0.10
α-pinene	0.92 ^a^ ± 0.01	0.93 ^a^ ± 0.01	0.55 ^b^ ± 0.08	0.55 ^b^ ± 0.14
*p*-cymene	0.76 ^ab^ ± 0.04	0.80 ^a^ ± 0.02	0.57 ^c^ ± 0.07	0.62 ^bc^ ± 0.06
d-limonene	0.84 ^a^ ± 0.04	0.87 ^a^ ± 0.01	0.55 ^a^ ± 0.07	0.59 ^a^ ± 0.24

Group A compounds share similarities in that they are less hydrophobic, more volatile compounds. These compounds have more affinity for the gelatine phase, and are unaffected by addition of sucrose. Table 4 shows that group A compounds have more affinity for the gelatine phase as the aroma distribution ratio is greater than 0.5 for these compounds. Release of less hydrophobic compounds such as ethyl acetate, seems to be less affected by matrix composition than more hydrophobic compounds (Seuvre, Philippe, Rochard, & Voilley, 2006). Release of these is not only determined by the properties of the compounds and interactions with solutes, but also by the properties of the matrix and is limited by diffusion in the matrices (Seuvre et al., 2006; Taylor et al., 2001). Previous research has shown that in gelatine based gels, there is a greater release of the less hydrophobic compounds compared to the more hydrophobic ones (Boland et al., 2004).

Group B (Table 4) includes compounds that have more affinity for the starch phase, with different functional groups. Some compounds (esters and linear aldehydes) are affected by addition of sucrose, where a significantly increased affinity for the starch phase is observed (P < 0.05), and other compounds (benzaldehyde, terpene alcohols and alcohols) are not (P > 0.05). The terpene alcohols geraniol and linalool are hydrophobic based on their Log P value and also less volatile, however the effect of sucrose on the distribution ratio follows the pattern of the less hydrophobic alcohols, rather than the more hydrophobic terpenes in group C (Table 4). This suggests that the hydroxyl group, and hence functional group of a volatile compound, is important in predicting its behaviour in food matrices. The presence of the hydroxyl group also prevents it interacting fully with hydrophobic pockets of proteins (Reiners et al., 2000), and linalool has also been shown to form inclusion complexes with starch (Lafarge, Bard, Breuvart, Doublier, & Cayot, 2008), therefore has strong affinity with the starch phase rather than protein. The position of a functional group may also impact interactions and binding, as shown by (Golovnya et al., 2001). Therefore many factors contribute to the extent to which aroma volatiles interact with a hydrocolloid based food matrix with high solute concentrations, and models to predict aroma volatile behaviour tend to be complex, such as the one produced by Friel et al. (2000) even in a simple aqueous solution.

Since the addition of sucrose between 40% and 60% causes disaggregation of polysaccharides (Fig. 2b, Table 5) (Kasapis et al., 2003), more free amylose and amylopectin are present in solution to form complexes, effectively increasing the potential for hydrophobic interactions and inclusion complexes to form (Arvisenet et al., 2002; Escher et al., 2000; van Ruth et al., 2003). Above 40% sucrose, gelatine forms a stronger network (Kasapis et al., 2003), thus a greater physical entrapment effect is observed for compounds, whereas a weaker polysaccharide network is formed.

For group C compounds, at concentrations below those required for the disaggregation effect, no significant difference (P > 0.05) between 20% sucrose and 0% sucrose was observed (Table 4) that do show a significant difference (P < 0.05) above 40% sucrose. These compounds share structural features in that a long hydrophobic chain or mainly hydrophobic portion is present, and have decreasing affinity for gelatine when sucrose is added above 40%. The length of hydrocarbon chain is important in determining the binding capacity and stability of amylose, since for shorter chain compounds such as ɗ-decalactone, a higher concentration is required for saturation of amylose compared to γ-decalactone (Heinemann et al., 2001). Similar effects are reported regarding chain length of esters binding to proteins (Reiners et al., 2000), which explains the groupings for esters other than ethyl decanoate. Ethyl decanoate and γ-decalactone therefore are expected to have more affinity for the starch phase yet show similar behaviour as the terpenes. These compounds share similarities in that their hydrophobic portions could pass a threshold of chain length or structure that forms strong stable interactions with the hydrophobic pockets of proteins (Reiners et al., 2000), compared to smaller esters.

Sucrose had a similar effect on the water activity of both phases and is equally distributed in each phase (Table 4) therefore the increase in affinity for the starch phase is not likely to do with the changes in water activity, rather the increased hydrophobic interactions. To test this hypothesis further, maltodextrin was added to a mixture with 0% sucrose, since amylose inclusion complexes consist of the aroma volatile binding in the hydrophobic cavity of or in between 6 and 8 glucose residues (Escher et al., 2000). A significantly lower distribution ratio was observed for many of the group B aroma compounds that were not significantly affected by sucrose. This suggests greater increased inclusion complex formation since there are more free helices, and also a threshold of helices available to interact has to be reached to have an effect on aroma distribution (data in supplementary material). Below the threshold, the free helices preferentially interact with the aldehydes and esters, and once a threshold of helices is reached, there is a retention effect of the other compounds previously unaffected by an increase in helices caused by sucrose addition. Maltodextrins have been suggested as flavour carriers as they have better flavour retention properties than native starches (Escher et al., 2000). A cooperative binding effect is observed as binding of a volatile compound may induce a change in amylose configuration from random coil to helix, to enhance further complexation with aroma (Yeo, Thompson, & Peterson, 2016).

Since retention of aldehydes by both starch (van Ruth et al., 2003) and proteins (Friel & Taylor, 2001) has been reported in literature, these results suggest that amylose-aroma interactions with the more hydrophobic aldehydes are stronger than the possible non-covalent interactions and Schiff base or condensation reactions between aroma and proteins.

### 3.5 Phase separated sucrose gels

A less aggregated and more dispersed starch network is also observed in the mixed hydrocolloid gel when sucrose is increased to 60% (Table 5). The aggregated starch network, phase separated from the clear gelatine network, forms a turbid gel at 0% sucrose. With increased levels of sucrose, this disaggregation and greater dispersion effect results in a loss of turbidity, as well as reduced water activity and also a longer dissolution time (Table 5). A lower water activity is expected as sucrose is hydrated with water, reducing the free water in the mixture. Since a less heterogeneous network structure is formed through stabilisation of the junction zones by sucrose, forming smaller and more junction zones, there is a decrease in turbidity (Nishinari et al., 1992; Normand et al., 2003). These turbidity and microstructure observations in this gelatine-starch matrix are in agreement with the study on gelatine and other polysaccharides (Kasapis et al., 2003).

**Table 5:** Effect of sucrose on properties of gelatine-starch gels. Letters in superscript indicate a significant difference between the samples on each row.

Sucrose (%w/v)	0	20	40	60
Turbidity				
Microstructure				
Water activity	0.996^a^	0.989^b^	0.976^c^	0.956^d^
Dissolution time (s)	30^a^	47^b^	58^c^	67^c^

Release rates of seven compounds were measured to represent different physicochemical properties and functional groups (Fig. 3). Increased sucrose levels resulted in a reduction in initial release rates of cymene, ethyl butyrate and butanal, the more volatile compounds in the mixture. These compounds all have stronger affinity for gelatine as discussed in section 3.4, and this result could be explained by both the increase in rigidity of gelatine (Kasapis et al., 2003) and by the increase in dissolution time for the gels (Table 5) as a result of increased sucrose.

Compounds that have more affinity for starch show mixed responses. The effect of sucrose on the microstructure could explain the results for the more volatile of these compounds that show an increase in release rate as a result of sucrose addition. Since a more dispersed starch network is formed, there is a greater surface area for release of the compound distributed in the starch phase, and hence a faster release is observed. However, linalool shows the opposite trend, which suggests other factors are involved and not just an effect of microstructure. These differences in behaviour in the presence of sucrose, and also compounds that show no obvious trends in change in release rates such as ethyl decanoate, has been explained by considering the changes in the mole fraction upon addition of a solute relative to the activity coefficient of the volatile (Friel et al., 2000; Voilley, Simatos, & Loncin, 1977). Sucrose-aroma interactions are considered to be unlikely as only hydrogen bonds can be formed, therefore the main effect of sucrose on aroma release is indirectly through the salting in or out effects (Friel et al., 2000; van Ruth & Roozen, 2000), and the different effects on protein and polysaccharide network formation (Kasapis et al., 2003).

## 4 Conclusions

Modifying the phase volumes of a gelatine-starch biphasic gel to achieve different microstructures and textures has no significant effect on aroma release *in vivo* (P > 0.05). Addition of sucrose to this mixture affected the microstructure as less aggregation of the polysaccharide was observed. In general, less hydrophobic and more volatile compounds are not significantly affected (P > 0.05) by sucrose concentration changes compared to the more hydrophobic, less volatile compounds. Aroma distribution and release vary depending on the physicochemical properties of the volatile.

When reducing solutes in a hydrocolloid matrix, whether for nutritional purposes or other reformulation purposes, it is important to consider the effects on the overall flavour balance of the reduced solute product. This study has shown the varied effects of increased sucrose concentrations in a protein-polysaccharide matrix on aroma distribution and aroma release. If a flavour mixture contains several key aroma compounds that are on the hydrophobic end of the scale, it is important to consider how changing the structure of the matrix affects release of these compounds, and more importantly perception of overall flavour of the altered product.

## CRediT authorship contribution statement

**Katy Su:** Investigation, Methodology, Validation, Formal analysis, Visualization, Writing - original draft, Writing - review & editing. **Marine Brunet:** Investigation, Methodology, Validation. **Daniel Festring:** Writing - review & editing, Supervision. **Charfedinne Ayed:** Formal analysis, Writing - review & editing. **Tim Foster:** Methodology, Supervision, Writing - review & editing. **Ian Fisk:** Funding acquisition, Supervision, Writing - review & editing.

## Declaration of competing interest

The authors declare that they have no known competing financial interests or personal relationships that could have appeared to influence the work reported in this paper.
