# Supplementary material for: Flavour distribution and release from gelatine-starch matrices
Source: Food Hydrocoll. 2021 Mar;112:106273. doi: 10.1016/j.foodhyd.2020.106273 (PMC7768189; doi:10.1016/j.foodhyd.2020.106273)
Supplement: Multimedia component 1 [file mmc1.docx]

**Supplementary material**

**Flavour distribution and release from gelatine-starch matrices**

Effect of maltodextrin at 5% and 10% on aroma distribution in gelatine-starch phase separated solutions. Different letters indicate significant differences for each compound.
